# Mammary Development and Breast Cancer: a Notch Perspective

**DOI:** 10.1007/s10911-021-09496-1

**Published:** 2021-08-10

**Authors:** Weizhen Chen, Wei Wei, Liya Yu, Zi Ye, Fujing Huang, Liyan Zhang, Shiqi Hu, Cheguo Cai

**Affiliations:** 1 Department of Orthopaedics, Frontier Science Center for Immunology and Metabolism, Medical Research Institute, Zhongnan Hospital of Wuhan University, Wuhan University, Wuhan, 430071 China; 2 DU-ANU Joint Science College, Shandong University, Weihai, 264200 China

**Keywords:** Notch signaling, Mammary development, Breast cancer, Stem cell

## Abstract

Mammary gland development primarily occurs postnatally, and this unique process is complex and regulated by systemic hormones and local growth factors. The mammary gland is also a highly dynamic organ that undergoes profound changes at puberty and during the reproductive cycle. These changes are driven by mammary stem cells (MaSCs). Breast cancer is one of the most common causes of cancer-related death in women. Cancer stem cells (CSCs) play prominent roles in tumor initiation, drug resistance, tumor recurrence, and metastasis. The highly conserved Notch signaling pathway functions as a key regulator of the niche mediating mammary organogenesis and breast neoplasia. In this review, we discuss mechanisms by which Notch contributes to breast carcinoma pathology and suggest potentials for therapeutic targeting of Notch in breast cancer. In summary, we provide a comprehensive overview of Notch functions in regulating MaSCs, mammary development, and breast cancer.

## Introduction

The mammary gland distinguishes mammals from all other animals. Its function is to produce and secrete milk in order to nourish offspring. Development of the mammary gland is unique: its main stages occur postnatally under the influence of steroid hormones. Throughout the lifespan of female mammals, the mammary gland may undergo multiple rounds of expansion and proliferation accompanied by structural and functional changes caused by pregnancy. Because of these features, the mammary gland is an ideal model for research into adult stem cell and organ development. Indeed, studies on mammary gland development have offered insights into mechanisms regulating cell fate specification, branching morphogenesis, and involution of functional organs. As such, the mouse mammary gland has been widely used to investigate how systemic hormones and local growth factors coordinate to control mammary development and breast cancer.

**Fig. 1:**
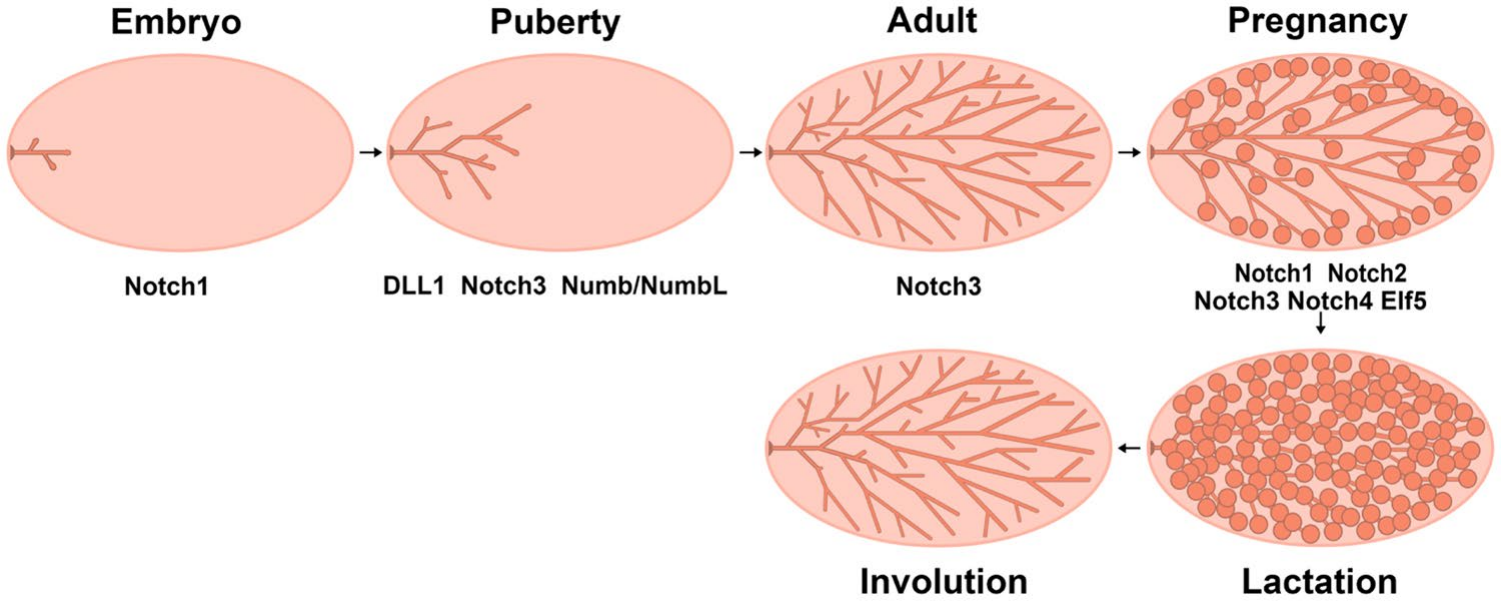
Diagram of postnatal mammary gland development. Mammary gland development involves four major stages: embryonic, pubertal, adult and reproductive. According to previous reports, Notch1 regulates epithelial cell differentiation during embryonic development. DLL1, Notch3 and Numb/NumbL affect mammary duct elongation and side branch formation at puberty. Besides, Notch3 regulates the formation of duct side branches at adult stages. During pregnancy, Notch1-4 could regulate alveolar cells formation and milk production. In addition, Elf5 could function during pregnancy via Notch signaling

Mammary gland development involves four major stages: embryonic, pubertal, adult and reproductive. During embryogenesis, the rudimentary ductal tree, which includes a primary duct and several secondary branches, is formed. This simple ductal system is still present at birth and remains largely quiescent during postnatal development. At puberty, estrogen and other system factors induce ductal elongation and branching, which results in formation of an elaborate epithelial ductal tree extending throughout the entire fat pad. In the adult mammary gland, ductal complexity increases through lateral branching in response to recurrent estrous cycles under progesterone stimulation. During pregnancy, progesterone stimulates the formation of alveolar clusters on small bore side branches. These secretory structures function to synthesize and secrete milk for the nourishment of offspring. After weaning, the mammary gland is remodeled, returning to the adulthood state [1]. Overall, mammary gland development is regulated by a complex network of systemic hormones and local growth factors. Interestingly, many of the pathways and processes that are deregulated in breast cancer are related to those controlling normal mammary gland development and remodeling (Fig. 1).

Breast cancer remains a major health problem and is currently a top biomedical research priority. Despite progress in early detection and clinical therapy, metastasis and drug resistance remain a formidable challenge. Extensive research has aimed to further understand breast carcinogenesis and to develop novel targeted therapies to improve patient survival outcomes.

In the past few years, dysregulation of Notch signaling has been recognized as a critical determinant of breast cancer initiation [2], progression [3], metastasis [4] and therapeutic resistance [5]. Here, we summarize major progress on the role of the Notch signaling pathway in the regulation of mammary stem cells (MaSCs) and gland development. We also provide an overview on how alterations in this pathway contribute to initiation, progression, metastasis, and therapy resistance of breast cancer. Finally, we discuss strategies for treating breast cancer by regulating the Notch pathway.

## The Mammalian Notch Signaling Pathway

**Fig. 2:**
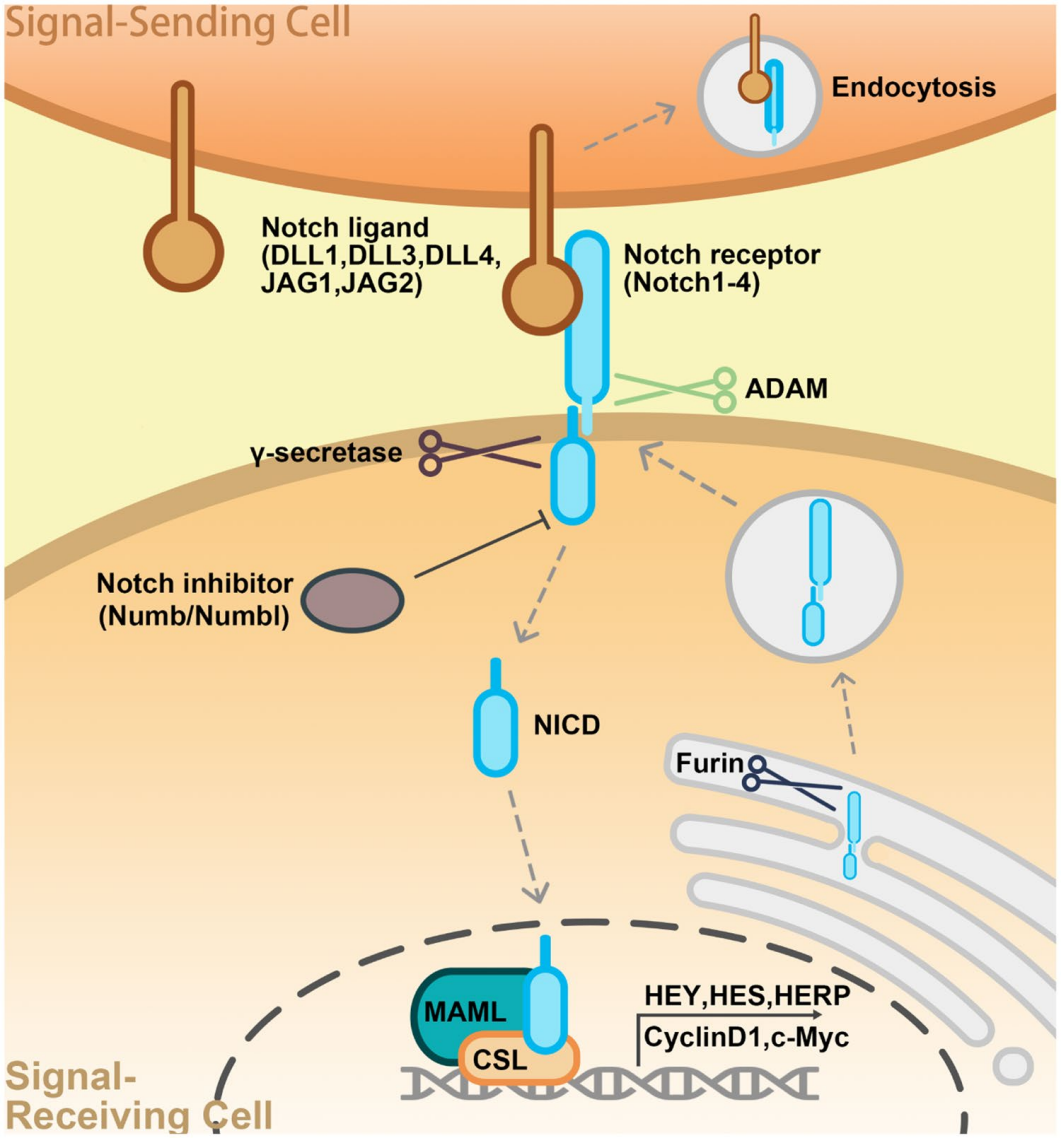
Canonical Notch signaling cascades in mammals. Notch ligands bind to the extracellular domain (NECD) of their receptor proteins, inducing receptor transactivation to expose the receptor transmembrane domain for ADAM/TACE metalloenzyme-mediated hydrolysis. NECD is pulled from the intracellular domain (NICD), and the NICD is cleaved by γ-secretase at an intracellular position to produce the free NICD fragment. The NICD is translocated from the cytoplasm to the nucleus, where it binds with CSL (CBF1/RBP-J/Su(H)/Lag-1) transcription factors to recruit a coactivator (MAML) and form a transcription complex that can activate the transcription of downstream genes (*HEY*, *HES*, *HERP*, *CyclinD1*, *c-Myc*, and others)

As a short-range signaling system, the Notch pathway plays a regulatory role in both signal-generating and signal-receiving cells [6]. Notch signal transduction occurs through direct contact between transmembrane ligands and receptors. In mammals, canonical Notch ligands include Delta-like (Dll)-1, -3, and -4, as well as Jagged (Jag)-1 and -2, while Notch receptor homologs include Notch-1, -2, -3, and -4 [7]. In the absence of a bound ligand, receptors are in an inactive state. Dll and Jag ligands bind to the Notch extracellular domain (NECD), pulling it towards the ligand-expressing cell, thereby exposing an S2 cleavage site within the negative regulatory region [8]. This site is then cleaved by ADAM/TACE metalloprotease. Ultimately, Notch extracellular sequences are endocytosed into the ligand-expressing cell. Subsequently, the intracellular portion of Notch together with attached transmembrane domain sequences and a small extracellular facing stump are cleaved again, this time by γ-secretase, which targets the transmembrane domain, thereby releasing a Notch intracellular domain fragment (NICD). Next, the NICD translocates to the nucleus, where it binds to a CSL (CBF1/RBP-J/Su(H)/Lag-1): MAML activation complex and induces transcription of target genes. The list of Notch targets includes several members of the *HES* and *HEY* gene families, which code for small bHLH transcriptional repressors. Thus, Notch activation is typically associated with upregulation of one or more *HES* and *HEY* genes. In some tissues, cell cycle regulators including *Cyclin D1* and *c-Myc* are Notch targets [9–11] (Fig. 2).

Notch, which has been studied for more than 100 years, is involved in regulating cell fate during development of many if not most tissues. In fact, Notch is a key regulator of stem cell maintenance [12], cell fate specification [13], and differentiation [14] during mammary gland development. Abnormal Notch activation and/or mutation of Notch genes has been linked to breast cancer [15]. Clinical studies have revealed an association between high-level expression of NOTCH1 and JAG1 in breast cancer and poor prognosis [3, 16]. In addition, NUMB, a negative regulator of Notch signaling, is expressed at a low level in 50% of breast tumors [17].

## Notch Signaling in Mammary Gland Development

### Notch Signaling Pathway Gene Expression in Mouse Mammary Gland

Notch pathway components (including ligands, receptors, and target genes) are differentially expressed across various mammary epithelial cells throughout the development of the mammary gland. Expression of Notch signal components has been primarily examined at the mRNA level. Among Notch ligands, *Dll1* is predominantly expressed in mammary basal cells and peaks during early involution. *Jagged1* is exclusively expressed in mammary luminal cells, and *Dll3* is expressed in mammary luminal and basal cells, with both of these reaching a peak during pregnancy and decreasing markedly during lactation. Other Notch ligands, such as *Jagged2* and *Dll4* are uniformly expressed at relatively low levels in mammary luminal and basal cells during both nulliparous periods and pregnancy. Among Notch receptors, *Notch1*, *-2*, and *-3* are predominantly expressed in mammary luminal cells of 5-week-old mice, and peak in early pregnancy, gradually decreasing during involution; *Notch4* is expressed in mammary luminal and basal cells at a low level. Among Notch target genes, *Hes1* begins expression in mammary epithelium cells at the prepubertal stage and reaches a peak in adulthood and early pregnancy; *Hes5* is stably expressed in the mammary gland throughout postnatal mammary development at a low level. *Hey1* and *Hey2* are both highly expressed in mammary luminal progenitor (CD61^+^) cells, though there are differences in the temporal patterns of *Hey1* and *Hey2* expression: expression of *Hey1* is high during pregnancy and early involution but low during lactation and late involution, while expression of *Hey2* is maintained at a high level throughout mammary development [18–20].

A few Notch signal components have been measured at the protein level in mouse epithelium. In the embryo, Notch1 is expressed in basal cells and can be used to identify unipotent stem cells [13]. At puberty, the Notch ligand Dll1 is expressed in myoepithelial cells, and Jagged1 is expressed in stromal cells around the terminal end bud (TEB); in adults, the expression of Dll1 is maintained in myoepithelial cells, while the expression of Jagged1 moved from stromal to myoepithelial cells; expression of Notch-1 and -4 is low in luminal and basal cells, while expression of Notch2 is high in stromal but low in epithelial cells, and expression of Notch3 is only found in luminal cells [21].

### Notch Signaling Pathway Functions in Mouse Mammary Gland Development

Notch signaling is implicated in MaSCs cell-fate determination. Notch1-expressing cells are unipotent stem cells with long-term plasticity in the mouse embryonic mammary gland. Expression of active *Notch1* can enable basal cells to acquire the characteristics of ER-negative luminal cells and promote the gradual movement of transformed cells to the duct lumen [13]. In addition, inhibition of Notch signaling by knockdown or disruption of *Cbf-1* [18, 22] could induce luminal to basal cell transdifferentiation with upregulation of *p63* as well as ectopic proliferation of MaSCs. Conversely, continuous expression of the activated form of Notch1 in MaSCs promoted luminal cell specification [18]. Similarly, knockout (KO) of *Numb/Numbl* (a Notch signaling inhibitor) dramatically reduced ductal elongation while enhancing TEB formation during puberty. Further mechanistic studies indicated that basal layer-specific genes were downregulated, while luminal layer-specific genes were upregulated in these knockout mice [23].

Notch signaling can also regulate the behavior of luminal progenitor cells. Lafkas et al*.* used a conditionally inducible Notch3-CreERT2^SAT^ transgenic mouse model to demonstrate that *Notch3* is highly expressed in luminal progenitor cells. A lineage tracing experiment revealed that Notch3 signaling limited the division and expansion of luminal progenitor cells and maintained them in a quiescent state [14]. A Notch3 KO mouse model exhibited defects in duct elongation and the formation of TEBs and side branches at puberty and adult stages, which were caused by a decrease in CCL2/CCR4 axis signaling [24]. In addition, overexpression of activated *Notch1* or *Notch3* also inhibited the proliferation of ductal and alveolar epithelial cells at puberty and early pregnancy. Mice with overexpression of activated *Notch1* or *Notch3* were unable to feed their pups because of decreased production of β-casein [25]. Cell ablation and long-term lineage tracing studies showed that Notch2-positive cells are required for formation of tertiary branches and alveolar clusters [26]. In addition, ectopic expression of *Notch4ICD* in MMTV-Int3 (Int3 = Notch4ICD) impaired ductal growth and differentiation, as well as secretory lobule formation during pregnancy [27–30]. The *Cbf-1* knockout suppressed abnormal lobular-alveolar development in mice with mammary-specific expression of activated Notch4 [31]. Similarly, *Elf5*, an inhibitor of the Notch pathway, is expressed primarily in luminal cells. The *Elf5*-null mouse mammary gland exhibits complete failure of alveologenesis during pregnancy, accompanied by inhibition of luminal progenitor cell differentiation [32]. Collectively, these data suggest that the canonical Notch/Cbf-1 pathway is necessary for luminal cell fate specification and maintenance, as well as control of differentiation in this lineage.

Notch signaling maintains normal mammary gland development through interaction with Wnt signaling. A recent study revealed that Dll1 KO mice showed reduced MaSCs number and impaired mammary ductal morphogenesis. Further study indicated Dll1^+^ MaSCs can increase Wnt ligand (Wnt3/10/16) expression by activating Notch2/3 signaling in stromal macrophages. In turn, Wnt signaling initiates a feedback loop to regulate activity of Dll1^+^ MaSCs [12]. The Notch and Wnt signaling pathways also interact in mammary epithelial cells to regulate normal mammary gland development. Studies have shown that suppression of the ratio of Wnt to Notch signaling within the mammary stem/progenitor cell compartment of parous mice was associated with a reduced proliferation potential [33] (Fig. 1).

### Notch Signaling Pathway Expression and Function in Human Mammary Epithelium

**Fig. 3:**
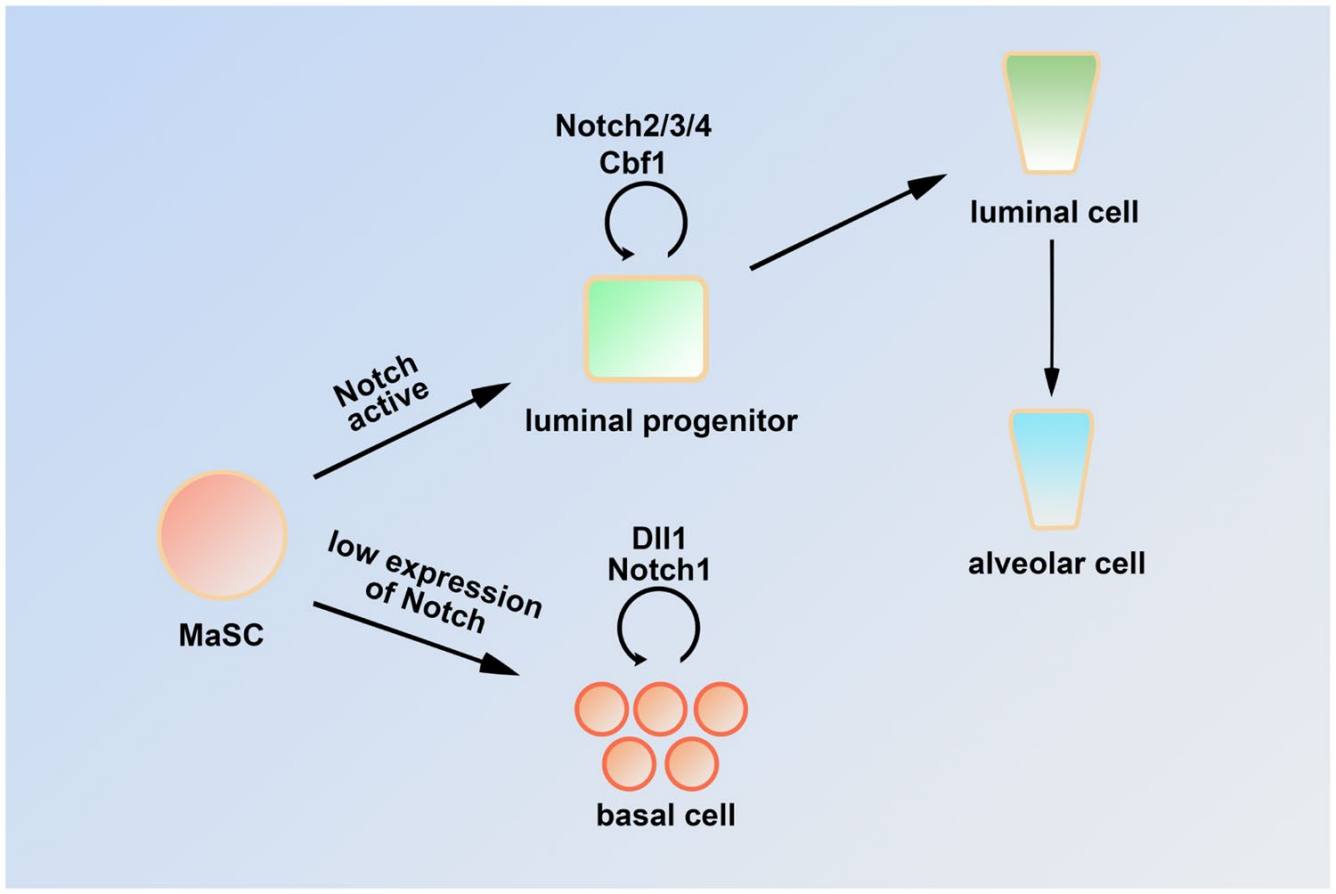
Model of the canonical Notch signaling pathway in mouse and human mammary gland epithelial cell development. The Notch pathway has dual functions in mammary gland epithelial cells. In basal cells, DLL1 and Notch1 are necessary to maintain the activity and self-renewal ability of MaSCs, and inhibition of Notch signaling leads to abnormal MaSCs expansion. In addition, Notch2/3/4 and Cbf1 could promote luminal progenitor cell differentiation towards the luminal cell lineage

The distribution of the Notch signaling pathway components in human breast epithelium was investigated by Raouf et al*.* [34]. Based on gene expression, human breast epithelial cells were divided into four distinct subpopulations: bipotent colony-forming cells (CFCs), luminal-restricted CFCs, mature luminal cells, and mature myoepithelial cells. Transcriptional profiling of each population showed that expression of *DLL1*, *JAG1*, and *JAG2* was higher in bipotent CFCs than in luminal-restricted CFCs. The expression of *NOTCH-1*, *-2,* and, most significantly, *NOTCH-3* was increased during luminal cell differentiation. Similarly, transcription of the target genes *HES1*, *HES6*, and *HEY1* was upregulated in luminal-restricted CFCs. In contrast, *NOTCH4* expression showed the opposite profile, with relatively high transcription levels in bipotent CFCs [34]. In situ hybridization also showed that *NOTCH3* was located to the luminal epithelium, while JAG1-positive cells were located within the basal layer [16]. Further functional study indicated that inhibition of Notch activity could induce myoepithelial colony formation ability and reduce the number of luminal colonies. Knockdown of NOTCH3 in the bipotent CFCs could reduce their ability to generate luminal cells, showing that NOTCH3 signaling is critical for breast progenitor cell differentiation in vitro [34]. Raouf et al*.* used immunohistochemistry to detect the location of Notch receptors and ligands proteins. The results showed that NOTCH-1 and -3 and their ligands, JAG-1 and -2, were most prominently expressed in the luminal epithelium of the normal human breast. Further functional study indicated that activation of RBP-Jk-dependent signaling could lead to normal human breast transformation, which could protect cells from drug-induced apoptosis [15]. Dontu et al*.* used an in vitro model to show that Notch activation promoted self-renewal of MaSCs and early progenitor cell proliferation. These effects were eliminated upon Notch signal inhibition [35]. Collectively, these results demonstrate that Notch signaling maintains the morphological structure of normal human epithelial cells (Fig. 3).

## Notch Signaling and Breast Carcinogenesis

### Notch Signaling and Primary Breast Carcinogenesis

Earlier studies have evaluated the significance of Notch signaling in primary breast epithelial carcinogenesis. In 1992, an activated mouse Notch-related Int-3 was expressed in a mouse model, leading to poor differentiation and hyper-proliferation of mammary gland epithelia [27]. In 1996, the same group confirmed that insertional mutation of the entire intracellular domain of the mouse Int3 protein also could induce abnormal differentiation, mammary dysplasia, and tumorigenesis [29]. In 2004, ectopic expression of human Notch4/Int3 protein in mice confirmed that those mice also could develop mammary tumors [2]. Hu et al*.* indicated that overexpression of intracellular domain activated alleles of *Notch- 1* or- *3* could also induce mammary gland transformation and tumorigenesis [25]. In addition, the Notch signal target, HeyL transgenic mice display accelerated mammary gland epithelial proliferation and develop mammary gland cancer through inhibition of the activity of TGFβ signaling [36]. An in vitro culture model further indicated that Notch signaling can suppress the apoptosis of human mammary epithelial cells [37]. NOTCH signaling is also essential for Wnt-induced tumorigenic conversion of primary human mammary epithelial cells [38]. All studies indicated that Notch signaling is crucial to primary mammary tumor development.

### Notch Signaling and Breast Ductal Carcinoma in Situ

Ductal carcinoma in situ (DCIS) is a pre-invasive malignant lesion that has increased markedly in incidence in recent decades. If DCIS is untreated, it will progress to invasive cancer in 30–50% of patients [39, 40]. Even after therapy, approximately 15–20% of patients with DCIS will experience recurrence within 10 years, at which time half of the recurrences are invasive [41]. To better understand its pathogenesis, Farnie et al*.* used the in vitro mammosphere culture technique to study the molecular properties of DCIS. Their results showed that NOTCH signaling is aberrantly activated, with high-level accumulation of *NICD* as well as the Notch target gene *HES1.* Treatment with Notch antagonists (DAPI) or Notch4 neutralizing antibodies could inhibit the formation of primary ductal carcinoma. Accumulation of NICD in DCIS was also associated with recurrence after surgery in clinical studies [42]. Pre-clinical human DCIS models further confirmed that combined Notch signaling inhibitor (DAPT) and Her2 inhibitors (lapatinib) could effectively inhibit DCIS acini growth and stem cell activity [43]. In addition, NOTCH3 can interact with HER2 to induce ductal carcinoma in situ, suggesting that combination therapy with anti-HER2 antibodies and Notch antagonists (such as GSIs) could be effective treatment for such lesions [44]. Those studies provide the possibility of targeting Notch to reduce recurrence rates of DCIS.

### Notch Signaling and Cancer Stem Cells

Cancer stem cells (CSCs) with the capacity for self-renewal can generate diverse differentiated tumor cell subpopulations. They are the origin of metastasis and highly resistant to therapeutic treatments. Their deregulation mechanisms differ from those of normal stem cells. Therefore, understanding the origin of CSCs and their deregulated pathways is important for tumor control [45, 46]. Previous reports have suggested that Notch signaling could promote self-renewal of CSCs [47–49]. Tumor stem cells expressing the Notch ligand Dll1 are linked to chemotherapy resistance in breast cancer. Pharmacological blocking of Dll1 can reactivate tumor cells sensitive to chemotherapy. Therefore, targeting of Dll1 combined with chemotherapy may be an effective strategy for the treatment of breast cancer chemoresistance [49]. NOTCH1 or DLL4 was shown to promote self-renewal of breast tumor stem cells by induced SIRT2-mediated deacetylation of ALDH1A1 at lysine 353 (K353), thereby activating it [50]. NOTCH4 can maintain quiescent mesenchymal-like CSCs by upregulation of SLUG and GAS1 [24]. The expression of *NUMB* is deficient or reduced in triple-negative breast cancer (TNBC), and low *NUMB* expression in CSCs is associated with reduced distant metastasis-free survival. Interestingly, low *NUMB* expression results in enhanced BRCA1-dependent tumor formation [17]. Similarly, the downregulation of NUMBL in tumor cells can also induce Notch activation, leading to an increase in epithelial to mesenchymal transition (EMT) and the cancer stem cell-like pool. In clinical breast tumor samples, NUMBL is downregulated, and the absence of NUMBL induces tumor cell chemoresistance for targeted therapy [51]. There are also other factors involved in regulation of CSCs via the Notch pathway. The NF-ĸB-JAG1 signaling axis regulates CSC populations in the basal-like subtype of breast cancer [52]. Lipid mediator sphingosine-1-phosphate (S1P) regulates CSCs populations via NICD/CSL/MAML complex-mediated gene transcription [53]. The Cargo protein MAP17 interacts with NUMB to overexpress the Notch pathway, leading to CSCs feature activation of tumor cells [46]. An actin-bundling protein, FASCIN, can mediate breast cancer chemoresistance and metastasis by maintaining the CSC pool stem cell-like phenotype and reversing EMT. The internal regulation mechanism is mediated by Notch signaling activation [54]. Additionally, NOTCH signaling mediates the stimulation role of estrogen on CSCs [55].

There is evidence that hypoxia promotes stem cell renewal in vitro as well as in vivo [56–58]. It therefore seems reasonable that stem cell survival is strictly connected with the hypoxia response at the molecular level. Previous studies indicated that Notch pathway factors mediate the relationship between stem cell survival and hypoxia response. In clinical breast cancer samples, both JAG2 and Notch signaling is upregulated at the hypoxic invasion front and JAG2 is correlated with metastasis-free survival of breast cancer patients. In tumor cells, hypoxia induces JAG2-mediated Notch signaling activation. Activated Notch further induces EMT and the self-renewal of cancer stem-like cells to promote tumor progression and metastasis [59]. In a hypoxic environment, the 66-kDa isoform of the SHC gene (p66Shc) is upregulated to regulate NOTCH3. The p66Shc/NOTCH3 interplay modulates self-renewal and hypoxia survival in breast cancer stem/progenitor cells [60]. In addition, IL-6 signaling also promotes a tumor hypoxia-resistant/invasive phenotype by triggering NOTCH3-expressing stem/progenitor cells [61].

### Notch Signaling and Triple-Negative Breast Cancer

TNBC constitutes 10–20% of all breast cancers and has a relatively poor prognosis. Because of the lack of estrogen receptor, progesterone receptor, and HER2 proteins expression, it cannot be effectively treated with current targeted therapies [62]. Therefore, understanding its molecular marker is important to overcome it. Previous studies have investigated the function of Notch signaling in TNBC where it is hyperactivated. Clinical analyses showed that *JAG1* [3, 16, 63, 64] as well as *NOTCH1* [16], *NOTCH3* [65], and *NOTCH4* [66] are expressed at high levels in TNBC and are associated with poor clinical prognosis. NOTCH3 can promote tumor cell self-renewal and aggressive tumor behavior; thus, targeting NOTCH3 to prevent breast cancer metastasis is clinically efficacious in breast cancer patients [65]. Tumors harbor numerous gene mutations. Stoeck et al*.* performed exome sequencing analysis to identify Notch mutations in various solid tumors, revealing that constitutive receptor activation induced by NOTCH1 and NOTCH2 mutations is limited to TNBC. A TNBC cell line with NOTCH1 rearrangement also exhibited high-level *N1ICD* accumulation and was sensitive to γ-secretase inhibitors (GSIs). In contrast, a cell line with NOTCH2 rearrangement was resistant to GSIs. Moreover, expression of the Notch target, *HES4*, was correlated with poor prognosis outcomes in TNBC patients [67]. In addition, NOTCH1 or NOTCH2 mutations can synergistically act with EZH2 to inhibit the tumor suppressor, PTEN transcription at the promoter in TNBC [68]. Currently, TNBC includes basal-like breast cancer (BLBC) [69]. In BLBC, activated Notch signaling was shown to promote expression of the inflammatory factors IL1B and CCL2 to recruit macrophages, further activating TGF-β-mediated signaling in tumor cells, thereby promoting the development of breast cancer [70]. NOTCH3 activation also drives tumor growth [71] and regulates stroma-mediated therapeutic resistance by expanding therapy-resistant tumor-initiating cells in BLBC [72]. Further, *LFNG*, a key determinant of NOTCH ligand specificity, is expressed at low levels in BLBC, and removal of *LFNG* from mouse breast tissue promoted cell proliferation and induced initiation of BLBC, accompanied by amplification of the MET/CAV locus. Moreover, these mutations can interact, resulting in the accumulation of NICD polypeptides which activate Notch signaling and enhance Met and Igf-1R signaling. Thus, combination therapy targeting Notch, MET, and IGF1R signaling may benefit BLBC patients with MET/CAV overexpression [21]. These studies provide a framework for the design of new treatment strategies based on key roles for JAG1, NOTCH1, NOTCH3, and NOTCH4 signaling in TNBC.

### Notch Signaling and ER-Positive/PR-Positive Breast Cancer

Consistent with its role in luminal cell fate specification, Notch signaling is critically involved in hormone receptor-positive breast cancers. Kumar et al*.* showed that *DLL1* is overexpressed in ERα-positive luminal breast cancer, where it correlates with poor prognosis. DLL1-mediated Notch signaling can promote tumor cell proliferation, angiogenesis, and tumor stem cell function. In that study, estrogen was shown to stabilize the DLL1 protein by inhibiting its proteasomal and lysosomal degradation [73]. In ERα-positive cells, estradiol can inhibit Notch activity and affect expression as well as cellular distribution of *NOTCH1*. Unfortunately, tamoxifen and raloxifene can reactivate Notch activity in ERα-positive breast cancer cells, leading to tamoxifen resistance [74]. This has led to the suggestion that anti-estrogen and anti-Notch combination therapy could prove beneficial for patients with ERα-positive disease. However, loss of Notch signaling can result in loss of ERα expression together with endocrine therapy-resistance [75]. In this regard, Sansone et al*.* have shown that NOTCH3 is involved in regulation of IL6-mediated hormone therapy resistance in breast cancer. Here, NOTCH3 renders tumors insensitive to hormone therapy by downregulating estrogen receptor expression [76]. In hormone-therapy resistant breast cancers, tamoxifen can also enhance stemness via JAG1-NOTCH4 signaling. Thus, despite clear indications of reciprocal interaction between ERα and multiple Notch receptor signaling pathways, suggestive of therapeutic potential [5, 77], this requires further nuanced investigation.

### Notch Signaling and HER2-Positive Breast Cancer

Although HER2-positive breast cancer has been successfully cured with target anti-HER2 agents [78, 79], a significant number of patients showed resistance, leading to disease recurrence and poor prognosis [80]. Therefore, development of a new molecular marker is vital for the treatment of patients that show resistance. Shah et al*.* found that pharmacologic inhibition of HER2 increases survival of CSCs in HER2 + breast cancer. This increased role was mediated by JAG1 upregulation. Thus, high-level *JAG1* accumulation in the cell membrane can be used to predict post-treatment response to anti-HER2 therapy [81]. In addition to JAG1, NOTCH1 also was confirmed to display upregulation in trastuzumab resistance, and inhibition of NOTCH1 could restore sensitivity to trastuzumab. In clinical trials, patients with HER2-positive breast cancers with high JAG1 or NOTCH1 expression show low overall survival. Therefore, inhibition of HER2 followed by inhibition of JAG1 or NOTCH1 may be more effective in preventing drug resistance and tumor progression than a simultaneous blockade [82–84].

### Notch Signaling and Breast Cancer Metastasis

**Fig. 4:**
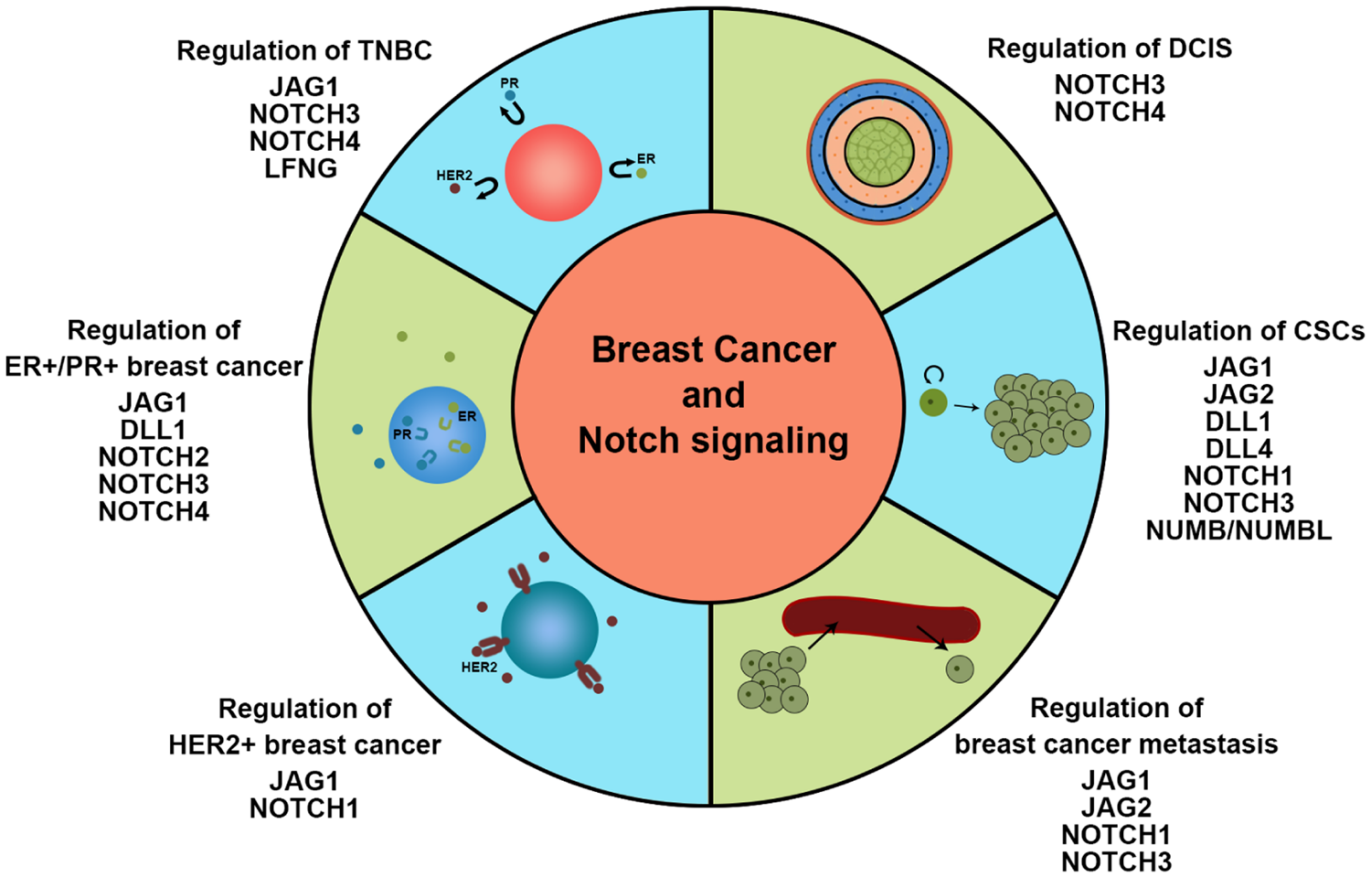
The cartoon schematically depicts the involvement of Notch pathway components in breast carcinogenesis

Notch signaling is also involved in metastatic dissemination of breast cancer, which is one of the major causes of breast cancer-associated mortality [85]. Activation of Notch by its ligand JAG1 can promote breast tumor growth and metastasis by inducing EMT. Regulation is mediated through Slug-induced repression of the cell–cell adhesion protein, E-cadherin [86]. Jag1 also mediate bone metastasis of breast cancer. Jag1 derived from tumor cells can promote maturation of osteoclasts and osteolytic bone metastasis of breast cancer by regulating release of IL6 from osteoblasts. Jag1 is also an effective downstream mediator of TGFβ, which is released during bone destruction. Disruption of Notch signaling with DAPT in stromal bone cells could reduce Jag1-mediated bone metastasis [87]. Activation by hypoxia of another Notch ligand, JAG2, is also correlated with metastasis-free survival of breast cancer patients, and may be a valuable prognostic marker for metastatic breast cancer [59]. Activation of the Notch receptor NOTCH1 could induce breast cancer metastasis by upregulating the EMT process, which is mediated by the pro-tumorigenic factor, CYR61 [88]. Interestingly, another receptor, NOTCH3, promotes bone metastasis of breast cancer but inhibits its lung metastasis. Zhang et al*.* confirmed that the TGFβ1-NOTCH3-JAG1 signaling axis mediated osteoblast-cancer cell interactions to promote the breast cancer bone metastasis. Knockdown of NOTCH3 could inhibit the bone metastasis process [89]. However, Lin et al*.* show that NOTCH3 activation suppresses the EMT of breast cancer, leading to inhibition of lung metastasis. Results of further studies indicated that the lung metastasis inhibition role of NOTCH3 was mediated by direct upregulation of GATA3 expression [90] (Fig. 4).

## Targeting the Notch Signaling Pathway in Breast Cancer

Given the substantial roles of Notch signaling in breast carcinogenesis, targeting Notch signaling inhibitors and monoclonal antibodies (mAbs) has been a focus of preclinical and early clinical research [91, 92]. Here, we discuss the currently known inhibitors and mAbs development process. NOTCH gene mutations have been identified in human breast cancer [93]. For example, Wang et al*.* showed that mutations in sequences coding for the PEST domains of NOTCH1, NOTCH2, and NOTCH3 have been found in TNBC. Cells with these mutations show elevated Notch signaling and are sensitive to PF-03084014 (a GSI Notch inhibitor) [94]. Simoes et al*.* demonstrated that a NOTCH4 signaling inhibitor RO4929097 (a distinct GSI) effectively inhibits resistance to antiestrogen therapy in hormone receptor-positive breast cancer [5]. Mamaeva et al*.* studied Notch signaling and the glycolytic phenotype in CSCs and found that DAPT-loaded partially glucosylated silica nanoparticles targeting CSCs effectively reduced the number of tumor stem cells [95]. In addition, both BXL0124 (a Gemini vitamin D analog) [96] and paeoniflorin (a major active ingredient in Chinese peony) [97] can block tumor cell proliferation by inhibiting Notch1 activation. As potent inhibitors of Notch signaling, BXL0124 and paeoniflorin may be used to treat BLBC. All small molecule Notch pathway inhibitors currently being investigated in phase I and II clinical trials target γ-Secretase (NCT01151449, NCT01071564, NCT01238133, NCT01217411, NCT01208441, NCT01158274, NCT02299635, NCT02338531, NCT01876251, NCT00106145, NCT00645333, NCT02784795, and NCT03422679). These inhibitors include R4929097, PF030814, and MK0752 (Table 1), which are being tested, either as monotherapies or in combination with other chemotherapeutic agents against a wide range of malignancies including TNBC, metastatic breast cancer, and advanced breast cancer. For example, in a phase I clinical trial, the combination of GSI R04929097 plus paclitaxel and carboplatin was used to treat stage II or stage III TNBC. In addition, NOTCH1 and Notch pathway inhibitors have been developed in clinical trials. In preclinical studies, mAbs have shown promise [98]. For example, mAbs has been used against human DLL4, MMGZ01 and H3L2 to disrupt DLL4-NOTCH1 signaling within tumor-associated endothelium. MMGZ01 and H3L2 both effected intratumoral angiogenesis with resultant tumor cell apoptosis [99, 100]. In addition, Sharma et al*.* developed a mAb against human NOTCH1 that inhibited MDA-MB-231 cell proliferation, induced apoptosis in these cells, and reduced their cancer stem cell-like phenotype. Thus, targeting individual Notch receptors with specific mAbs is a potential therapeutic strategy to reduce the BCSC-like subpopulation [101] (Table 1).

## Conclusions

Recent studies on the Notch signaling pathway have made rapid progress in delineating mechanisms responsible for regulation of self-renewal and differentiation of MaSCs, mammary development, BCSC maintenance, metastasis, and drug resistance in breast cancer. Along with these advances, several potential drugs, such as GSIs and anti-ligand and anti-receptor antibodies, have been developed to treat aggressive subtypes of breast cancer. Although these drugs show some therapeutic effects, they also have strong side effects. Therefore, more research is required before these compounds can be applied clinically. Future studies should focus more heavily on fine-tuning the function of Notch and/or Notch signaling in regulation of various breast cancer subtypes and further explore the crosstalk between Notch and other pathways in these contexts. Studies on such crosstalk may lead to the development of novel and effective combination therapies.

**Table 1:** Notch pathway inhibitors in preclinical studies and in current clinical trials of breast cancer

Classification	Compound and combination or intervention	Target	Breast cancer type	Clinicaltrials.gov identifier	Phase	State	References
Experimental antagonists	PF03084014	$\gamma$-Secretase	TNBC with PEST domain mutation of Notch1, Notch2, and Notch3	/	/	Preclinical	[94]
	RO4929097	$\gamma$-Secretase	ER+, resistant breast cancer	/	/	Preclinical	[5]
	Mesoporous silica nanoparticles with DAPT	$\gamma$-Secretase	Cancer stem cells	/	/	Preclinical	[95]
	BXL0124	Notch1	Basal-like breast cancer cell lines	/	/	Preclinical	[96]
	Paeoniflorin	Notch1	Breast cancer cell lines	/	/	Pre clinical	[97]
Neutralizing antibody	DLL4 antibody	DLL4	Triple-negative breast cancer	/	/	Preclinical	[99, 100]
	Notchi1 antibody	Notch1	Triple-negative breast cancer	/	/	Preclinical	[101]
Small molecules in current clinical trials	RO4929097	$\gamma$-Secretase	Advanced, metastatic, or recurrent triple negative	NCT01151449	II	Terminated	https://clinicaltrials.gov/ct2/home
	RO4929097 plus vismodegib	$\gamma$-Secretase	Breast cancer that is metastatic or cannot be surgically resected	NCT01071564	I	Terminated	
	RO4929097 plus paclitaxel and carboplatin	$\gamma$-Secretase	Stage II or stage III triple-negative breast cancer	NCT01238133	I	Terminated	
Small molecules in current clinical trials	RO4929097 and whole-brain radiation therapy or stereotactic radiosurgery	$\gamma$-Secretase	Brain metastases from breast cancer	NCT01217411	I	Terminated	https://clinicaltrials.gov/ct2/home
	RO4929097 plus letrozole	$\gamma$-Secretase	Postmenopausal women with hormone receptor-positive stage II or stage III breast cancer	NCT01208441	I	Terminated	
	RO4929097 plus capecitabine	$\gamma$-Secretase	Refractor solid tumors	NCT01158274	I	Completed	
	PF03084014	$\gamma$-Secretase	Advanced breast cancer with or without Notch alterations	NCT02299635	II	Terminated	
	PF03084014	$\gamma$-Secretase	Chemoresistant triple-negative breast cancer	NCT02338531	II	Withdrawn	
	PF03084014 plus docetaxel	$\gamma$-Secretase	Metastatic breast cancer	NCT01876251	I	Terminated	
	MK0752	$\gamma$-Secretase	Advanced breast cancer	NCT00106145	I	Completed	
	MK0752 plus docetaxel and pegfilgrastim	$\gamma$-Secretase	Metastatic breast cancer	NCT00645333	I/II	Completed	
	LY3039478	Notch 1	Advanced or metastatic solid tumors including breast cancer	NCT02784795	I	Completed	
	CB-103	Notch pathway	Advanced or metastatic solid tumors including breast cancer	NCT03422679	I/II	Recruiting	

## References

[1] Fu NY, et al. Stem Cells and the Differentiation Hierarchy in Mammary Gland Development. Physiol Rev. 2019;100. 10.1152/physrev.00040.2018 31539305

[2] Raafat A (2004). Mammary development and tumorigenesis in mice expressing a truncated human Notch4/Int3 intracellular domain (h-Int3sh). Oncogene.

[3] Dickson BC (2007). High-level JAG1 mRNA and protein predict poor outcome in breast cancer. Mod Pathol.

[4] Shao S (2015). Notch1 signaling regulates the epithelial-mesenchymal transition and invasion of breast cancer in a Slug-dependent manner. Mol Cancer.

[5] Simoes BM (2015). Anti-estrogen Resistance in Human Breast Tumors Is Driven by JAG1-NOTCH4-Dependent Cancer Stem Cell Activity. Cell Rep.

[6] Artavanis-Tsakonas S, et al. Notch Signaling Cell Fate Control and Signal Integration in Development. Science. 1999;284. 10.1126/science.284.5415.770 10221902

[7] D'Souza B (2008). The many facets of Notch ligands. Oncogene.

[8] Langridge PD, et al. Epsin-Dependent Ligand Endocytosis Activates Notch by Force. Cell. 2017;171(6):1383–96 e12. 10.1016/j.cell.2017.10.048 PMC6219616 29195077

[9] Henrique D, et al. Mechanisms of Notch signaling: a simple logic deployed in time and space. Development. 2019;146(3). 10.1242/dev.172148 30709911

[10] Andersson ER, Sandberg R, Lendahl U (2011). Notch signaling: simplicity in design, versatility in function. Development.

[11] Siebel C, Lendahl U (2017). Notch Signaling in Development, Tissue Homeostasis, and Disease. Physiol Rev.

[12] Chakrabarti R, et al. Notch ligand Dll1 mediates cross-talk between mammary stem cells and the macrophageal niche. Science. 2018;360(6396). 10.1126/science.aan4153 PMC7881440 29773667

[13] Lilja AM (2018). Clonal analysis of Notch1-expressing cells reveals the existence of unipotent stem cells that retain long-term plasticity in the embryonic mammary gland. Nat Cell Biol.

[14] Lafkas D (2013). Notch3 marks clonogenic mammary luminal progenitor cells in vivo. J Cell Biol.

[15] Stylianou S, Clarke RB, Brennan K (2006). Aberrant activation of notch signaling in human breast cancer. Cancer Res.

[16] Reedijk M (2005). High-level coexpression of JAG1 and NOTCH1 is observed in human breast cancer and is associated with poor overall survival. Cancer Res.

[17] Rennstam K (2010). Numb protein expression correlates with a basal-like phenotype and cancer stem cell markers in primary breast cancer. Breast Cancer Res Treat.

[18] Bouras T (2008). Notch signaling regulates mammary stem cell function and luminal cell-fate commitment. Cell Stem Cell.

[19] Raafat A (2011). Expression of Notch receptors, ligands, and target genes during development of the mouse mammary gland. J Cell Physiol.

[20] Pal B (2017). Construction of developmental lineage relationships in the mouse mammary gland by single-cell RNA profiling. Nat Commun.

[21] Xu K (2012). Lunatic fringe deficiency cooperates with the Met/Caveolin gene amplicon to induce basal-like breast cancer. Cancer Cell.

[22] Buono KD (2006). The canonical Notch/RBP-J signaling pathway controls the balance of cell lineages in mammary epithelium during pregnancy. Dev Biol.

[23] Zhang Y (2016). Numb and Numbl act to determine mammary myoepithelial cell fate, maintain epithelial identity, and support lactogenesis. FASEB J.

[24] Zhou L (2020). NOTCH4 maintains quiescent mesenchymal-like breast cancer stem cells via transcriptionally activating SLUG and GAS1 in triple-negative breast cancer. Theranostics.

[25] Hu C (2006). Overexpression of activated murine Notch1 and Notch3 in transgenic mice blocks mammary gland development and induces mammary tumors. Am J Pathol.

[26] Sale S, Lafkas D, Artavanis-Tsakonas S (2013). Notch2 genetic fate mapping reveals two previously unrecognized mammary epithelial lineages. Nat Cell Biol.

[27] Jhappan C (1992). Expression of an activated Notch-related int3 transgene interferes with cell differentiation and induces neoplastic transformation in mammary and salivary glands. Genes Dev.

[28] Smith GH, et al. Constitutive expression of a Truncated INT3 Gene in mouse mammary epithelium impairs differentiation and functional development. Cell Growth Differ. 1995;6:563–77. 7544153

[29] Gallahan D (1996). Expression of a Truncated int3 gene in developing secretory mammary epthelium specifically retards lobular dofferentiation resulting in tumorigenesis. Cancer Res.

[30] Gallahan D (1997). The mouse mammary tumor associated gene INT3 is a unique member of the NOTCH gene family NOTCH4. Oncogene.

[31] Raafat A (2009). Rbpj conditional knockout reveals distinct functions of Notch4/Int3 in mammary gland development and tumorigenesis. Oncogene.

[32] Chakrabarti R (2012). Elf5 regulates mammary gland stem/progenitor cell fate by influencing notch signaling. Stem Cells.

[33] Meier-Abt F (2013). Parity induces differentiation and reduces Wnt/Notch signaling ratio and proliferation potential of basal stem/progenitor cells isolated from mouse mammary epithelium. Breast Cancer Res.

[34] Raouf A (2008). Transcriptome analysis of the normal human mammary cell commitment and differentiation process. Cell Stem Cell.

[35] Dontu G, et al. Role of Notch signaling in cell-fate determination of human mammary stem-progenitor cells. Breast Cancer Res. 2004;6:R605–R615. 10.1186/bcr920 PMC1064073 15535842

[36] Han L (2014). The Notch pathway inhibits TGFbeta signaling in breast cancer through HEYL-mediated crosstalk. Cancer Res.

[37] Meurette O (2009). Notch activation induces Akt signaling via an autocrine loop to prevent apoptosis in breast epithelial cells. Cancer Res.

[38] Ayyanan A (2006). Increased Wnt signaling triggers oncogenic conversion of human breast epithelial cells by a Notch-dependent mechanism. Proc Natl Acad Sci U S A.

[39] Page DL (1995). Continued local recurrence of carcinoma 15–25 years after a diagnosis of low grade ductal carcinoma in situ of the breast treated only by biopsy. Cancer.

[40] Leonard GD, Swain SM (2004). Ductal carcinoma in situ, complexities and challenges. J Natl Cancer Inst.

[41] Sakorafas GH, Farley DR, Peros G (2008). Recent advances and current controversies in the management of DCIS of the breast. Cancer Treat Rev.

[42] Farnie G (2007). Novel cell culture technique for primary ductal carcinoma in situ: role of Notch and epidermal growth factor receptor signaling pathways. J Natl Cancer Inst.

[43] Farnie G, et al. Combined inhibition of ErbB1/2 and Notch receptors effectively targets breast ductal carcinoma in situ (DCIS) stem/progenitor cell activity regardless of ErbB2 status. PLoS One. 2013;8(2):e56840. 10.1371/journal.pone.0056840 PMC3572946 23457626

[44] Pradeep CR (2012). Modeling ductal carcinoma in situ: a HER2-Notch3 collaboration enables luminal filling. Oncogene.

[45] Dalerba P, Cho RW, Clarke MF (2007). Cancer stem cells: models and concepts. Annu Rev Med.

[46] Garcia-Heredia JM (2017). The Cargo Protein MAP17 (PDZK1IP1) Regulates the Cancer Stem Cell Pool Activating the Notch Pathway by Abducting NUMB. Clin Cancer Res.

[47] Harrison H (2010). Breast cancer stem cells: something out of notching?. Cancer Res.

[48] Harrison H (2010). Regulation of breast cancer stem cell activity by signaling through the Notch4 receptor. Cancer Res.

[49] Kumar S (2021). Dll1(+) quiescent tumor stem cells drive chemoresistance in breast cancer through NF-kappaB survival pathway. Nat Commun.

[50] Zhao D (2014). NOTCH-induced aldehyde dehydrogenase 1A1 deacetylation promotes breast cancer stem cells. J Clin Invest.

[51] García-Heredia JM, et al. Numb like (NumbL) downregulation increases tumorigenicity, cancer stem cell-like properties and resistance to chemotherapy. Oncotarget. 2016;7. 10.18632/oncotarget.11553 PMC5325389 27613838

[52] Yamamoto M (2013). NF-kappaB non-cell-autonomously regulates cancer stem cell populations in the basal-like breast cancer subtype. Nat Commun.

[53] Hirata N (2014). Sphingosine-1-phosphate promotes expansion of cancer stem cells via S1PR3 by a ligand-independent Notch activation. Nat Commun.

[54] Barnawi R (2016). Fascin Is Critical for the Maintenance of Breast Cancer Stem Cell Pool Predominantly via the Activation of the Notch Self-Renewal Pathway. Stem Cells.

[55] Harrison H (2013). Oestrogen increases the activity of oestrogen receptor negative breast cancer stem cells through paracrine EGFR and Notch signalling. Breast Cancer Res.

[56] Ramirez-Bergeron DL, et al. Hypoxia Inducible Factor and the Development of Stem Cells of the Cardiovascular System. Stem Cells. 2001;19:279–86. 10.1634/stemcells.19-4-279 11463947

[57] Cejudo-Martin P, Johnson RS (2005). A new notch in the HIF belt: how hypoxia impacts differentiation. Dev Cell.

[58] Covello KL (2006). HIF-2alpha regulates Oct-4: effects of hypoxia on stem cell function, embryonic development, and tumor growth. Genes Dev.

[59] Xing F (2011). Hypoxia-induced Jagged2 promotes breast cancer metastasis and self-renewal of cancer stem-like cells. Oncogene.

[60] Sansone P (2007). p66Shc/Notch-3 interplay controls self-renewal and hypoxia survival in human stem/progenitor cells of the mammary gland expanded in vitro as mammospheres. Stem Cells.

[61] Sansone P (2007). IL-6 triggers malignant features in mammospheres from human ductal breast carcinoma and normal mammary gland. J Clin Invest.

[62] Speiser JJ, Ersahin C, Osipo C (2013). The functional role of Notch signaling in triple-negative breast cancer. Vitam Horm.

[63] Reedijk M (2008). JAG1 expression is associated with a basal phenotype and recurrence in lymph node-negative breast cancer. Breast Cancer Res Treat.

[64] Cohen B (2010). Cyclin D1 is a direct target of JAG1-mediated Notch signaling in breast cancer. Breast Cancer Res Treat.

[65] Leontovich AA (2018). NOTCH3 expression is linked to breast cancer seeding and distant metastasis. Breast Cancer Res.

[66] Nagamatsu I, et al. Notch4 is a potential therapeutic target for triple-negative breast cancer. Anticancer Res. 2014;34:69–80. 24403446

[67] Stoeck A (2014). Discovery of biomarkers predictive of GSI response in triple-negative breast cancer and adenoid cystic carcinoma. Cancer Discov.

[68] Pappas K (2021). NOTCH and EZH2 collaborate to repress PTEN expression in breast cancer. Commun Biol.

[69] Lehmann BD (2011). Identification of human triple-negative breast cancer subtypes and preclinical models for selection of targeted therapies. J Clin Invest.

[70] Shen Q (2017). Notch Shapes the Innate Immunophenotype in Breast Cancer. Cancer Discov.

[71] Choy L (2017). Constitutive NOTCH3 Signaling Promotes the Growth of Basal Breast Cancers. Cancer Res.

[72] Boelens MC (2014). Exosome transfer from stromal to breast cancer cells regulates therapy resistance pathways. Cell.

[73] Kumar S (2019). Estrogen-dependent DLL1-mediated Notch signaling promotes luminal breast cancer. Oncogene.

[74] Rizzo P (2008). Cross-talk between notch and the estrogen receptor in breast cancer suggests novel therapeutic approaches. Cancer Res.

[75] Buckley NE (2013). BRCA1 is a key regulator of breast differentiation through activation of Notch signalling with implications for anti-endocrine treatment of breast cancers. Nucleic Acids Res.

[76] Sansone P (2016). Self-renewal of CD133(hi) cells by IL6/Notch3 signalling regulates endocrine resistance in metastatic breast cancer. Nat Commun.

[77] Haughian JM (2012). Maintenance of hormone responsiveness in luminal breast cancers by suppression of Notch. Proc Natl Acad Sci U S A.

[78] Carter P, et al. Humanization of an anti-p185HER2 antibody for human cancer therapy. Proc Natl Acad Sci USA. 1992;89:4285–89. 10.1073/pnas.89.10.4285 PMC49066 1350088

[79] Nahta R (2007). Lapatinib induces apoptosis in trastuzumab-resistant breast cancer cells: effects on insulin-like growth factor I signaling. Mol Cancer Ther.

[80] Vogel CL (2002). Efficacy and Safety of Trastuzumab as a Single Agent in First Line Treatment of HER2Overexpressing Metastatic Breast Cancer. J Clin Oncol.

[81] Shah D (2018). Inhibition of HER2 Increases JAGGED1-dependent Breast Cancer Stem Cells: Role for Membrane JAGGED1. Clin Cancer Res.

[82] Osipo C (2008). ErbB-2 inhibition activates Notch-1 and sensitizes breast cancer cells to a gamma-secretase inhibitor. Oncogene.

[83] Pandya K (2011). Targeting both Notch and ErbB-2 signalling pathways is required for prevention of ErbB-2-positive breast tumour recurrence. Br J Cancer.

[84] Baker A (2018). Notch-1-PTEN-ERK1/2 signaling axis promotes HER2+ breast cancer cell proliferation and stem cell survival. Oncogene.

[85] Zhang Y (2019). Notch and breast cancer metastasis: Current knowledge, new sights and targeted therapy. Oncol Lett.

[86] Leong KG (2007). Jagged1-mediated Notch activation induces epithelial-to-mesenchymal transition through Slug-induced repression of E-cadherin. J Exp Med.

[87] Sethi N (2011). Tumor-derived JAGGED1 promotes osteolytic bone metastasis of breast cancer by engaging notch signaling in bone cells. Cancer Cell.

[88] Ilhan M, et al. Pro-metastatic functions of Notch signaling is mediated by CYR61 in breast cells. Eur J Cell Biol. 2020;99(2–3):151070. 10.1016/j.ejcb.2020.151070 32005345

[89] Zhang Z (2010). Notch3 in human breast cancer cell lines regulates osteoblast-cancer cell interactions and osteolytic bone metastasis. Am J Pathol.

[90] Lin HY (2018). Notch3 inhibits epithelial-mesenchymal transition in breast cancer via a novel mechanism, upregulation of GATA-3 expression. Oncogenesis.

[91] Takebe N, Nguyen D, Yang SX (2014). Targeting notch signaling pathway in cancer: clinical development advances and challenges. Pharmacol Ther.

[92] Locatelli M, Curigliano G (2017). Notch inhibitors and their role in the treatment of triple negative breast cancer: promises and failures. Curr Opin Oncol.

[93] Robinson DR (2011). Functionally recurrent rearrangements of the MAST kinase and Notch gene families in breast cancer. Nat Med.

[94] Wang K (2015). PEST domain mutations in Notch receptors comprise an oncogenic driver segment in triple-negative breast cancer sensitive to a gamma-secretase inhibitor. Clin Cancer Res.

[95] Mamaeva V (2016). Inhibiting Notch Activity in Breast Cancer Stem Cells by Glucose Functionalized Nanoparticles Carrying gamma-secretase Inhibitors. Mol Ther.

[96] So JY (2015). HES1-mediated inhibition of Notch1 signaling by a Gemini vitamin D analog leads to decreased CD44(+)/CD24(-/low) tumor-initiating subpopulation in basal-like breast cancer. J Steroid Biochem Mol Biol.

[97] Zhang Q (2016). Paeoniflorin inhibits proliferation and invasion of breast cancer cells through suppressing Notch-1 signaling pathway. Biomed Pharmacother.

[98] Wu Y (2010). Therapeutic antibody targeting of individual Notch receptors. Nature.

[99] Xu Z (2016). MMGZ01, an anti-DLL4 monoclonal antibody, promotes nonfunctional vessels and inhibits breast tumor growth. Cancer Lett.

[100] Jia X (2016). A humanized anti-DLL4 antibody promotes dysfunctional angiogenesis and inhibits breast tumor growth. Sci Rep.

[101] Sharma A (2012). A monoclonal antibody against human Notch1 ligand-binding domain depletes subpopulation of putative breast cancer stem-like cells. Mol Cancer Ther.

